# Reliable and Rapid Robotic Assessment of Wrist Proprioception Using a Gauge Position Matching Paradigm

**DOI:** 10.3389/fnhum.2016.00316

**Published:** 2016-06-29

**Authors:** Mike D. Rinderknecht, Werner L. Popp, Olivier Lambercy, Roger Gassert

**Affiliations:** Rehabilitation Engineering Laboratory, Department of Health Sciences and Technology, Institute of Robotics and Intelligent Systems, ETH ZurichZurich, Switzerland

**Keywords:** wrist robot, wrist proprioception, reliability, quantitative measurements, psychophysics, proprioceptive testing

## Abstract

Quantitative assessments of position sense are essential for the investigation of proprioception, as well as for diagnosis, prognosis and treatment planning for patients with somatosensory deficits. Despite the development and use of various paradigms and robotic tools, their clinimetric properties are often poorly evaluated and reported. A proper evaluation of the latter is essential to compare results between different studies and to identify the influence of possible confounds on outcome measures. The aim of the present study was to perform a comprehensive evaluation of a rapid robotic assessment of wrist proprioception using a passive gauge position matching task. Thirty-two healthy subjects undertook six test-retests of proprioception of the right wrist on two different days. The constant error (CE) was 0.87°, the absolute error (AE) was 5.87°, the variable error (VE) was 4.59° and the total variability (E) was 6.83° in average for the angles presented in the range from 10° to 30°. The intraclass correlation analysis provided an excellent reliability for CE (0.75), good reliability for AE (0.68) and E (0.68), and fair reliability for VE (0.54). Tripling the assessment length had negligible effects on the reliabilities. Additional analysis revealed significant trends of larger overestimation (constant errors), as well as larger absolute and variable errors with increased flexion angles. No proprioceptive learning occurred, despite increased familiarity with the task, which was reflected in significantly decreased assessment duration by 30%. In conclusion, the proposed automated assessment can provide sensitive and reliable information on proprioceptive function of the wrist with an administration time of around 2.5 min, demonstrating the potential for its application in research or clinical settings. Moreover, this study highlights the importance of reporting the complete set of errors (CE, AE, VE, and E) in a matching experiment for the identification of trends and subsequent interpretation of results.

## 1. Introduction

Assessment of proprioception after neurological injuries and diseases has received increased attention, as there is growing evidence that somatosensory impairment leads to a poor prognosis for functional recovery after neurological injuries in patients with severe and persistent somatosensory dysfunction, such as after stroke (Kusoffsky et al., 1982; Feys et al., 2000; Han et al., 2002; Abela et al., 2012). This may be a consequence of the fact that proprioception is essential for the generation or correction of coordinated movements (Hasan, 1992; Sober and Sabes, 2003; Butler et al., 2004; Konczak et al., 2009) and critical for fine movements of the upper limb, e.g., aiming, reaching and grasping. Proprioception is thus of high importance in activities of daily living (Jeannerod et al., 1984; Ghez et al., 1990; Gentilucci et al., 1994; Carey, 1995; Sarlegna and Sainburg, 2009).

Proprioception is commonly divided into limb or joint position sense (the sense of stationary position) and kinaesthesia (the sense of limb movement) (Gilman, 2002). Mechanisms underlying the proprioceptive system, including the exact contribution of the different receptors to different aspects of proprioception, as well as processing at the spinal, subcortical and cortical level, are complex and not yet fully understood (Proske and Gandevia, 2012).

Accurate and sensitive assessments of proprioception are required, not only to investigate and understand the sense of proprioception and the effect of aging, but also for diagnosis, prognosis and treatment planning for patients with somatosensory deficits (Pumpa et al., 2015). As it is unarguable that there is no single measure of proprioception, it is not evident how to best quantify proprioception. There exist very few clinically accepted and used tests for proprioception, e.g., the static and dynamic up-down test (Lincoln et al., 1991; Gilman, 2002), a similar dual joint position test (Beckmann et al., 2013), positional mimicry and finger finding (Lincoln et al., 1991). Despite their specific advantages, such as being simple and quick to administer, these assessments are largely subjective, lack standardized protocols and suffer from large variability due to manual administration, resulting in poor inter-rater reliability (Lincoln et al., 1991, 1998; Winward et al., 1999). As a consequence of their dichotomous or ordinal scales, they are not precise and lack resolution, and are thus considered good for screening patients, but not for assessing functional improvements (Hillier et al., 2015). In accordance with these limitations, a cross-sectional survey of occupational therapists and physiotherapists reported that more than half agreed that current methods of assessing somatosensation should be improved (Pumpa et al., 2015).

Over the past years, more quantitative assessment concepts to investigate different aspects of proprioception have been proposed. Some use the combination of simple passive apparatuses restraining the movements to specific planes and matching paradigms with protractor scales (Carey et al., 1996; Wycherley et al., 2005; Schmidt et al., 2013), or size discrimination tasks by grasping spherical objects (Kalisch et al., 2012), and thus provide quantitative outcome measures. The number of robotic approaches to assess proprioception has also increased over the last few years, as they can take advantage of the control and sensing capabilities of robotic technology (Scott and Dukelow, 2011) to address requirements for an optimal assessment, such as high resolution, high reproducibility, and good control over stimuli. Different techniques have been reported in the literature (Han et al., 2015), such as threshold detection of passive motion (Kokmen et al., 1978; Wright et al., 2011; Ingemanson et al., 2015) and displacement perturbations (Simo et al., 2014; Bourke et al., 2015), joint position matching and reproduction (Ferrell et al., 1992; Lönn et al., 2000a,b; Adamo et al., 2007, 2009; Juul-Kristensen et al., 2008; Adamo and Martin, 2009; Dukelow et al., 2010, 2012; Gay et al., 2010; Squeri et al., 2011; Semrau et al., 2013; Herter et al., 2014; Nomura and Ito, 2014), and difference thresholds tracking methods (Lambercy et al., 2011; Rinderknecht et al., 2014; Cappello et al., 2015).

Even though a vast range of different paradigms for the assessment of proprioceptive function have been developed, of which some use expensive robotic devices or lengthy experimental protocols, many of those approaches constitute research-oriented assessments and are difficult to apply in clinical settings (Hillier et al., 2015). Furthermore, clinimetric properties, such as reliability, precision, feasibility and clinical utility, which are essential for establishing a new assessment, are often either poorly evaluated and reported, or not reported at all (for a review see Hillier et al., 2015).

The primary aim of the present study was to evaluate the test-retest reliability of a rapid robotic assessment of wrist proprioception using a passive gauge position matching task based on the Wrist Position Sense Test (WPST) from Carey et al. (1996) in young healthy subjects undertaking six test-retests. This gauge position matching approach was chosen because of its simplicity and low risk for confounds such as motor function or memory. We hypothesized that by using robotic technology in combination with this paradigm it is possible to achieve a high reliability through better reproducibility of stimuli and increase clinical utility by decreasing assessment time, as the stimulation and error recording process can be automated. The secondary aim was to investigate effects of the stimulus amplitude (i.e., presented angle), perceptual learning, and sex on the matching errors. These evaluations will reveal whether this specific robotic approach, based on gauge position matching, is suitable for assessing proprioception—potentially in a clinical setting.

## 2. Materials and methods

### 2.1. Subjects

A total of 32 healthy subjects participated in the study (age mean ± SD: 22.5 ± 2.6 years, 11 male and 21 female). Only right handed subjects were included. Handedness was assessed with the Edinburgh Handedness Inventory (Oldfield, 1971). The laterality index was larger or equal to 60 for all subjects (mean ± SD: 85.4 ± 13.2). Exclusion criteria were any somatosensory or motor deficits affecting normal wrist and hand function, or any history of neurological or wrist injury. All participating subjects had either normal or corrected-to-normal vision. All subjects gave written informed consent in accordance with the Declaration of Helsinki prior to participating in the experiment. The study was approved by the institutional ethics committee of the ETH Zurich (EK 2015-N-03).

### 2.2. Apparatus

**Figure 1:**
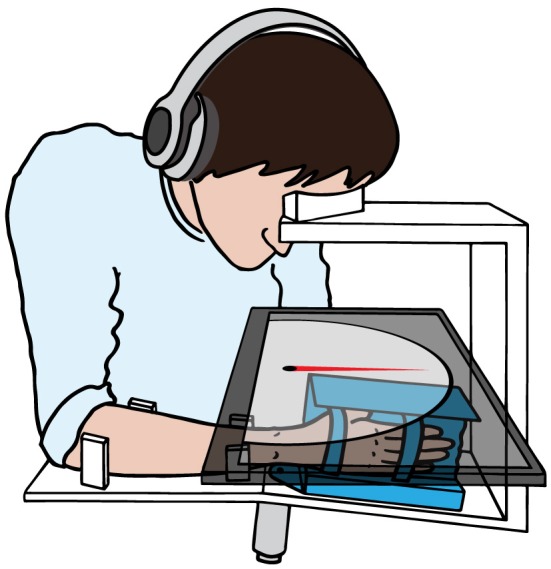
**Simplified schematic of the one degree-of-freedom apparatus used for the proprioception assessment**. The motor (gray) actuates the handle (blue) in wrist flexion-extension direction. A touchscreen (semitransparent dark gray) is placed over the wrist interface, in direct line of sight, occluding the tested wrist, hand and part of the forearm from vision. With the non-assessed hand, the subject aligns the gauge (red) on the touchscreen with the perceived wrist position. During the whole assessment, the head rests on the support frame and the subject hears white noise through headphones.

The assessment of wrist joint proprioception was performed with the ReFlex (Figure 1), a one degree-of-freedom robotic wrist interface (Chapuis et al., 2010). The ReFlex is capable of providing well-controlled and reproducible passive flexion-extension movements to the right wrist with a direct-drive brushed DC motor (RE65, Maxon Motor, Sachseln, Switzerland). The angular position is measured with a high-resolution optical encoder fixed to the motor shaft (R158, 1 million counts/rev, Gurley Precision Instruments, Troy, NY, USA) allowing for a good position and velocity resolution at high sampling rates during fast wrist movements. The ReFlex is controlled at 1 kHz by a target PC running LabVIEW RealTime 13.0 (National Instruments, Austin, TX, USA).

The visual interface is displayed on a touchscreen PC running Windows 7 with LabVIEW 2013 (National Instruments, Austin, TX, USA). The touchscreen is mounted horizontally above the tested wrist, such that the perceived wrist position can be indicated by the subject by aligning a displayed angular gauge indicator with the perceived orientation of the hand. This touchscreen allows at the same time to prevent the subject from seeing the tested wrist, hand and part of the forearm. The hand was attached to the handle by two Velcro straps, with the wrist joint, as well as all finger joints (distal interphalangeal, proximal interphalangeal, and metacarpophalangeal joints) aligned in one line—with exception of the thumb. To reduce visual parallax errors when aligning the gauge to the wrist position, a non-adjustable head support frame was mounted on top of the touchscreen ensuring reproducible head positions across subjects and sessions.

### 2.3. Experimental protocol

Subjects sat to the left of the device, and the device was adjusted for a snug fit and comfortable position of the forearm. The hand was strapped to the handle after ensuring an optimal alignment of the wrist joint and the motor axis. After placing the touchscreen on the frame on top of the hand, the subjects placed their foreheads on the head support to ensure visual alignment of the wrist joint and the gauge indicator. Subjects were asked to relax their limb during the assessment. White noise was played over headphones during the whole assessment in order to avoid auditory cues and mask any noise emitted by the motor. Only the proprioception of the right wrist joint was assessed, as the apparatus was specifically designed for the right wrist.

In every trial of the matching task the robotic device passively moved the handle from the resting position (0° flexion) to a specific flexion angle in 1 s using a minimum jerk trajectory (Hogan, 1984). As the movement duration was constant, the peak velocity was directly related to the extent of the flexion angle. Once the subject provided feedback on the perceived angle by adjusting the gauge indicator on the touchscreen, the device moved the handle back to the resting position with the same minimum jerk trajectory and the gauge indicator jumped back to the zero position. The gauge indicator could be manipulated by clicking directly on the touchscreen (and the gauge would immediately jump to this position) or by dragging the gauge to the desired position. Subjects had no time constraint to provide feedback and received no feedback on the accuracy of their response. In total, 21 angles (integer values in the range of 10° to 30° flexion) were each presented once in random order.

Each subject participated in two sessions on different days (from 1 to 34 days between sessions, mean ± SD: 7.4 ± 7.5 days). As it was assumed that the proprioceptive function of healthy young subjects remains stable over time, a more relaxed scheduling of the sessions was allowed to facilitate the subject recruitment process. Each session consisted of three consecutive assessments (3 × 21 trials) with a 1 min break between the assessments, resulting in a total of six test-retests.

### 2.4. Outcome measures

For completeness and to compare to other studies in the literature, we report constant error (*CE* = average error), absolute error (*AE* = average absolute error), variable error (*VE* = standard deviation of errors) and total variability (*E* = root mean square of errors) in degrees as proprioceptive outcome measures. The error is calculated as reported angle minus presented angle. Following this convention, a positive *CE* represents an overestimation of the wrist flexion angle, whereas a negative *CE* represents an underestimation. While the implementations of *CE*, *AE*, and *E* follow the standard definitions (Schmidt and Lee, 2011), the *VE* was implemented as the standard deviation of errors across all the presented angles, as each angle was presented only once and the classical definition would result in a non-zero *VE* for zero error. The proposed definition of *VE* also represents the variability in the error distribution between the trials, respectively angles. An additional outcome measure was the required administration time of the assessment, which is important for potential application in a clinical setting.

### 2.5. Data analysis

The test-retest reliability was calculated based on the intraclass correlation coefficient *ICC* (2, 1) (two-way layout with random effects for absolute agreement) (Shrout and Fleiss, 1979). Its 95% confidence interval (CI), the standard error of measurement *SEM* and the smallest real difference *SRD* (in the literature sometimes referred to as minimal detectable change *MDC*) were calculated according to Lexell and Downham (2005) and de Vet et al. (2006). Additionally, the test-retest reliability when pooling the three measurements of each session (i.e., averaging proprioceptive outcome measures of three measurements) was calculated to explore by how much the reliability could be improved by tripling the total number of trials.

The relationship between the presented angle and the proprioceptive outcome measures was analyzed by fitting errors of each subject (averaged for each presented angle over six measurements) with a linear function using ordinary least squares. Statistical significance was tested by comparing slopes to zero using one-sample *t*-tests, respectively Wilcoxon signed-ranks test for not normally distributed data.

In order to identify whether the errors and the assessment duration changed from measurement to measurement, non-parametric Friedman tests and *post-hoc* paired Wilcoxon signed-rank tests, or paired *t*-tests in case of normally distributed differences, with a Šidák-correction applied for multiple comparisons were performed. Additionally, to test whether a relationship between subject performance and inter-session time span existed, Pearson correlations were computed for each proprioceptive outcome measure using the inter-session time span in days and the mean of the three measurements of each session.

Potential effects of sex on the proprioceptive outcome measures were investigated by performing Wilcoxon rank-sum tests on the outcome measures *CE*, *AE*, *VE*, and *E* after averaging the six measurements.

Significance levels were set to α = 0.05. Probability values *p* < 0.05 and *p* < 0.01 are marked as ^*^ and ^*^^*^. Descriptive statistics are reported as mean ± SD, unless otherwise stated. All statistical analyses were performed in MATLAB R2014a (MathWorks, Natick, MA, USA).

## 3. Results

**Figure 2:**
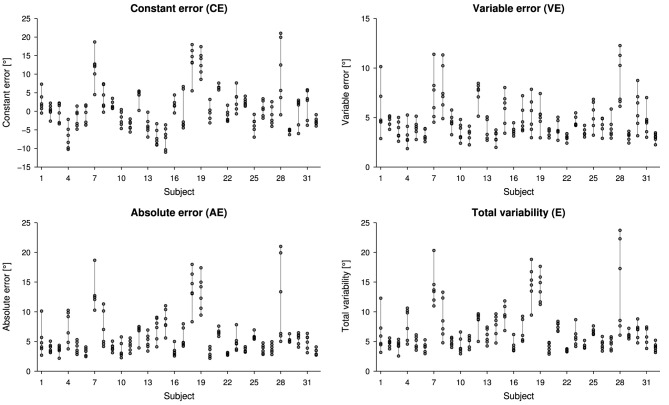
**Scatter plots illustrating the intra- and inter-subject variability for the outcome measures constant error (***CE***), absolute error (***AE***), variable error (***VE***) and total variability (*E*)**. Each measurement is represented by one circle.

Overall, proprioceptive outcome measures resulted in 0.87° ± 5.43° for *CE*, 5.87° ± 3.08° for *AE*, 4.59° ± 1.53° for *VE* and 6.83° ± 3.27° for *E*, considering all six measurements for each subject. Individual proprioceptive outcome measures for the six measurements are presented in Figure 2.

The reliabilities for the outcome measures *CE*, *AE*, *VE*, and *E* were between 0.54 and 0.75 for a single measurement, and differed negligibly when three measurements (M1–3 and M4–6, respectively) were pooled (Table 1). The *SEM* characterizing the measurement variability and the *SRD* for evaluating changes are listed in Table 1 for all four outcome measures.

**Table 1:** **Summary of the reliability analysis (intraclass correlation coefficients and confidence intervals) for a single measurement and for three pooled measurements (M1–3 vs. M4–6), as well as standard error of measurement (***SEM***) and smallest real difference (***SRD***) for a single measurement**.

	***r* [CI]**	**pooled *r* [CI]**	***SEM***	***SRD***
*CE*	0.75 [0.63, 0.85]	0.78 [0.60, 0.89]	3.09°	8.56°
*AE*	0.68 [0.55, 0.80]	0.65 [0.39, 0.81]	2.05°	5.68°
*VE*	0.54 [0.39, 0.69]	0.56 [0.26, 0.76]	1.33°	3.69°
*E*	0.68 [0.55, 0.80]	0.65 [0.39, 0.81]	2.17°	6.02°

**Figure 3:**
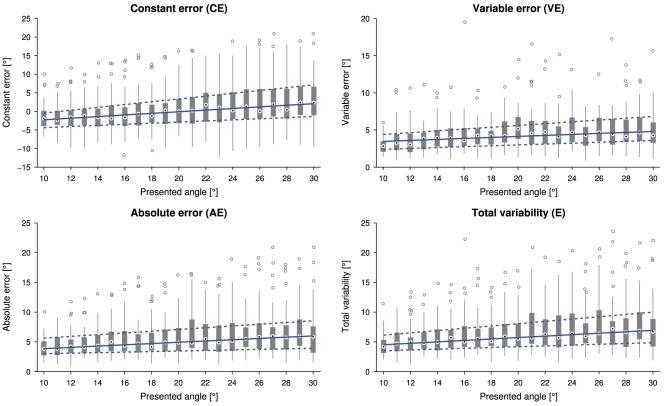
**Tukey box plots for the outcome measures constant error (***CE***), absolute error (***AE***), variable error (***VE***) and total variability (***E***) as a function of the presented angle (32 subjects, i.e., data points, per presented angle)**. Errors were individually averaged across six measurements for each subject and presented angle. The solid and dashed lines indicate a linear fit through the medians and lower and upper quartiles, respectively.

The linear relationship between the presented wrist angle and the outcome measures is shown in Figure 3. The slopes of the linear fits for *CE* (0.25±0.30), *AE* (0.14±0.20), *VE* (0.09±0.12) and *E* (0.16±0.21) were statistically significantly higher than zero [*CE*: *t*_(31)_ = 4.774, *p* < 0.0001, *AE*: *Z* = 3.6, *p* < 0.001, *VE t*_(31)_ = 4.207, *p* < 0.001 and *E*: *Z* = 3.7, *p* < 0.001].

**Figure 4:**
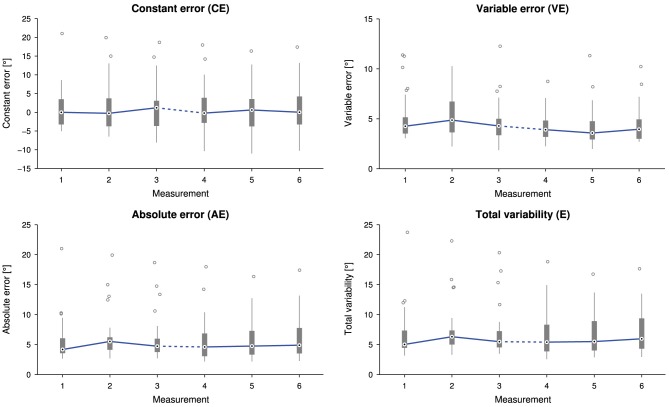
**Tukey box plots for the outcome measures constant error (***CE***), absolute error (***AE***), variable error (***VE***) and total variability (***E***) as a function of the measurement (32 subjects, i.e., data points, per measurement)**. Errors were individually averaged across 21 presented angles for each subject and measurement. The solid line connects the medians. The dashed line indicates a longer time interval between the two sessions (M1–3 and M4–6).

No correlation between the time span between the two sessions and a change in any of the proprioceptive outcome measures was found [*CE*: *r*_(30)_ = 0.01, *p* = 0.96, *AE*: *r*_(30)_ = 0.05, *p* = 0.77, *VE*: *r*_(30)_ = 0.10, *p* = 0.60, and *E*: *r*_(30)_ = 0.07, *p* = 0.69]. There was neither a statistically significant difference in *CE* [$X_{\left(5\right)}^{2}=1.946,p=0.857$], nor in *AE* [$X_{\left(5\right)}^{2}=9.750,p=0.083$], nor in *E* [$X_{\left(5\right)}^{2}=9.286,p=0.098$] depending on the measurement time point. Despite a statistically significant difference in *VE* depending on the measurement time point, $X_{\left(5\right)}^{2}=16.250,p=0.006$, *post-hoc* tests did not reveal any significant difference between paired measurement comparisons (Figure 4).

**Figure 5:**
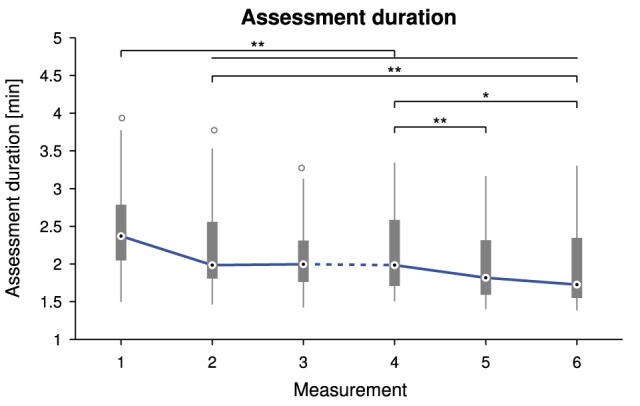
**Tukey box plots for the assessment duration as a function of the measurement time point**. The solid line connects the medians. The dashed line indicates a longer time interval between the two sessions (M1–3 and M4–6). Probability values *p* < 0.05 and *p* < 0.01 are marked as ^*^ and ^**^.

**Figure 6:**
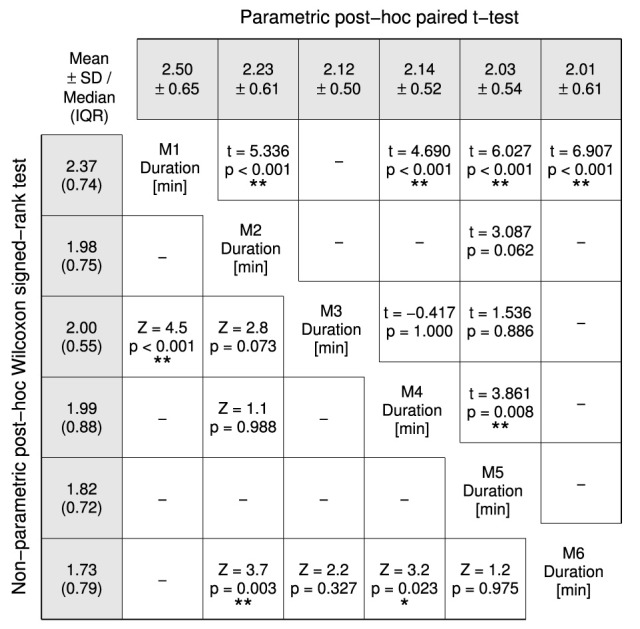
*****Post-hoc*** tests for the Friedman test comparing the assessment durations of the six measurements (M1–6)**. Non-parametric test results (*Z* statistic, *p*-value) are displayed in the lower-left half and parametric test results (*t*(31) statistic, *p*-value) in the upper-right half. A Šidák-correction was applied to all *post-hoc* tests. The gray-shaded fields list mean ± SD in the top row and median (inter quartile range) in the left column. Probability values *p* < 0.05 and *p* < 0.01 are marked as ^*^ and ^**^.

Overall average assessment duration was 2.2 ± 0.5 min, ranging from 1.4 to 3.9 min. There was a statistically significant difference in assessment duration depending on the measurement time point, $X_{\left(5\right)}^{2}$ = 66.125, *p* < 0.0001. Tukey box plots of the six measurements visualize the decreasing trend in Figure 5. Detailed descriptive statistics and *post-hoc* tests are grouped in Figure 6.

Statistical testing revealed no significant differences between the male and female subjects for any of the four proprioceptive outcome measures—neither for *CE* (*Z* = −0.6, *p* = 0.526, male: 0.22° ± 5.07°, female: 1.21° ± 5.70°), nor *AE* (*Z* = −1.0, *p* = 0.341, male: 5.42° ± 3.23°, female: 6.11° ± 3.04°), nor *VE* (*Z* = −0.0, *p* = 0.968, male: 4.35° ± 1.00°, female: 4.71° ± 1.75°), nor *E* (*Z* = −0.8, *p* = 0.405, male: 6.33° ± 3.26°, female: 7.09° ± 3.32°).

## 4. Discussion

In this study we evaluated an automated gauge position matching task using a robotic setup to assess wrist proprioception with regards to reliability, administration time, as well as effects of the stimulus amplitude (i.e., presented angle), perceptual learning, and sex on the matching errors. The proprioceptive outcome measures of the assessment consisted of the constant error, absolute error, variable error and total variability.

With a group average of 5.87° ± 3.08° for the absolute error, the obtained results correspond well to the scores of the WPST (6.1° ± 1.8°) in healthy subjects with an age range from 23 to 77 years (Carey et al., 1996) and another matching study using passive movement reproductions of passively presented movements with healthy subjects with a similar age range from 20 to 65 years (4.9 ± 2.9°) (Gay et al., 2010). Normative data from healthy control subjects is required for an enhanced diagnosis of proprioceptive deficits in clinical testing. As such, different studies have introduced percentiles describing healthy performance for different proprioceptive assessment paradigms (Carey et al., 1996; Dukelow et al., 2010; Semrau et al., 2013; Herter et al., 2014; Simo et al., 2014). This requires paradigm-specific studies with large sample sizes and age-matched subjects for an accurate model of healthy performance. In such a normative study comparing gauge position matching performance of 50 healthy subjects with 50 stroke patients, Carey et al. (1996) introduced the 100th percentile criterion for abnormality at an absolute average error of 11° (with 6.2–15.8° being the zone of uncertainty). In our study, two subjects with average absolute errors below the zone of uncertainty presented one measurement marginally above 11°. Four additional subjects had an average absolute error within the zone of uncertainty (averages from 11.9 to 13.9°). These four subjects also showed large variations between the different measurements, meaning that this outcome could result from general fatigue, inattention or lack of motivation, as they did not show an overall trend of decreasing performance across the six measurements. However, it was not possible to determine the exact cause for large errors based on the data or experimenter's observation. As we believe that such outliers are to be expected in any psychophysical experiment, and as their exclusion does not affect the general results of the present experiment in a significant way, they were considered in the data analysis and the results are presented including all subjects. The parameters of agreement (*SEM* and *SRD*) were in a similar range as for the WPST presented by Carey et al. (1996) and are less dependent on the heterogeneity of the sample compared to the reliability (de Vet et al., 2006). Based on the *SRD* it can be verified whether a clinical intervention generated a change between pre- and post-assessment, which is not the result of measurement error. Furthermore, this parameter is essential to determine if the assessment is capable of detecting clinically relevant changes (de Vet et al., 2006).

### 4.1. Good to excellent reliability for absolute error, constant error and total variability

The intraclass correlation analysis based on six test-retests provided an excellent reliability for the constant error, good reliability for the absolute error, as well as total variability, and fair reliability for the variable error, according to the general reliability recommendations (excellent: >0.75, fair to good: 0.4–0.75, poor: < 0.4) by Fleiss (1999). This suggests that the constant error, the absolute error and the total variability may not suffer from high intra-subject variability, and could be used as outcome measures for proprioception reflecting bias and extent of error, respectively. In return, the variable error could well represent proprioceptive acuity, as it reflects the limitation of information transfer due to noise (Clark et al., 1995). As visible in Figure 2, the variable errors show a large intra-subject variability compared to the inter-subject variability. Thus, it is not advisable to use the variable error as a meaningful outcome measure for subject performance consistency in young healthy subjects. Whether this result is also true for the assessment of patients with proprioceptive impairments, has to be established in a similar study with this specific population. It should be noted, that despite the total variability being explained equally by bias and response variability—in contrast to the absolute error, which doe not adequately present the variable error—(Henry, 1975), the reliability for the both the absolute error and the total variability are identical in this sample. As a matter of fact, there is a strong correlation between the two outcome measures.

The original study evaluating the manual WPST reported reliability coefficients for the absolute error of 0.92 and 0.88 when comparing sessions 1, 2, and 2, 3, respectively, in a population of 35 stroke patients (Carey et al., 1996). These reliability coefficients are higher compared to the value found in the present study, but this may result directly from a higher inter-subject variability of the stroke sample, independently of whether robotic technology was used for a more reproducible stimulus control. Two other studies assessing wrist proprioception using different paradigms also reported test-retest measurements in healthy subjects (Gay et al., 2010; Cappello et al., 2015), however, it is not clear how these results can be compared due to lack of detailed information on methodologies or results. There have been few other studies investigating reliability of proprioceptive assessments at more proximal joints (Lönn et al., 2000a; Juul-Kristensen et al., 2008; Dukelow et al., 2010; Simo et al., 2014) and at the level of the hand (Wycherley et al., 2005; Kalisch et al., 2012) showing reliabilities ranging from 0.007 to 0.92. However, all these reliability studies used rather small sample sizes of less than thirty subjects, although it has been recommended that the sample size should be at least 30 for test-retest reliability studies (Hopkins, 2000). Therefore, the reported reliabilities should be treated with caution—especially if confidence intervals are not reported. Nevertheless, it is interesting to note that, in a reliability study (15 stroke patients and 7 healthy subjects) using whole-limb proprioception assessments, the systematic error (comparable to the absolute error of our gauge position matching paradigm) showed lower reliability than the variability (comparable to the variable error) (Dukelow et al., 2010), which is opposite to our results. However, this study evaluated the inter-rater reliability and used an interval of a few minutes between the two measurements. Since such a short interval may not capture full intra-subject variability and may not prevent other confounding factors, such as recall bias, it is suggested to use longer intervals (but short enough so patients do not change on the measured construct) for evaluating test-retest reliability (Streiner and Norman, 2008). Thus, the reported inter-rater reliability may not be representative for the test-retest reliability. In addition, another study (26 healthy subjects) on active ipsilateral remembered elbow matching indicated good reliability for absolute errors and poor reliability for variable errors (Juul-Kristensen et al., 2008). Thus, it may be concluded that these inconclusive results arise due to the use of different paradigms, variability within assessed subject groups or small sample sizes.

Tripling the total number of trials from 21 to 63 (by pooling the three consecutive assessments of the same session, as if it was only one, but longer, assessment) has a negligible effect on the reliability of the four proprioceptive outcome measures. One could hypothesize that a longer assessment with three times as much data would lead to more powerful estimates and higher test-retest reliability by reducing estimate variability. In contrast to this hypothesis, these results suggest that a short assessment with 21 trials already provides a representative estimation of the subject's proprioceptive wrist function and that there is no major information and precision loss compared to a longer assessment. This is essential, as a short assessment duration is required for a potential application in a clinical setting. Nevertheless, it should also be added that the 95% confidence interval of the reliability is in this case rather large to determine small but significant improvements of reliability. Through a better control of external factors, such as motivation, fatigue, sleep or preceding physical activity, reliability may be improved, however these factors can be very difficult to control in a non-experimental setting.

### 4.2. Systematic overestimation and larger variability for larger angles

The analysis of the influence of stimulus amplitude (i.e., presented angle) on the errors showed significant and systematic trends toward larger absolute and variable errors, and higher total variability for larger flexion movements, as well as a trend toward overestimation (constant error). Using a three-dimensional geometric reconstruction of the setup, an assumed viewpoint offset of about 15 mm in the horizontal plane due to different head positions could result in an underestimation for small angles and overestimation of large angles of up to 1°. Despite parallax errors being a strong potential confound, they seem not to account for the complete effect visible in Figure 3. As a matter of fact, the error dependence on movement extent has to our knowledge not been extensively studied in such a gauge position matching task, and results of studies using similar matching paradigms with movement replications are somewhat inconsistent: For larger movements, there is some literature reporting greater absolute errors (Stelmach and Walsh, 1972; Roy and Kelso, 1977; Goble et al., 2006; Adamo et al., 2007) and increased variable errors (Roy and Kelso, 1977; Choi et al., 1995; Djupsjobacka and Domkin, 2005; Goble and Brown, 2008; Goble et al., 2009, 2010), i.e., decreased position matching acuity. The latter is suggested to depend on integration of movement-related information (Choi et al., 1995; Djupsjobacka and Domkin, 2005). While these studies are in line with our results, others showed no effects on the variable error (Marteniuk et al., 1972) or the absolute error (Marteniuk et al., 1972; Scott Kelso, 1977; Goble and Brown, 2008). Since the absolute error and the total variability are combinations of the constant error and the variable error (Schutz and Roy, 1973; Henry, 1975), it is important to report and analyze the underlying constant error for a meaningful interpretation of results. Analyses of the constant error in studies involving remembered active matching in shoulder and elbow joints yielded mostly small overshooting or undershooting for large angles and greater overshooting for small angles (Marteniuk et al., 1972; Marteniuk, 1973; Roy and Kelso, 1977; Scott Kelso, 1977; Lönn et al., 2000b; Goble et al., 2009). Few studies show no effect and general overshooting, respectively, general undershooting (Stelmach and Walsh, 1972; Goble and Brown, 2008; Goble et al., 2010). The tendency to overshoot short movements and undershoot long movements has been described as the range effect (Pepper and Herman, 1970). However, this effect is well studied and known to apply to movement replications, but has not been shown in gauge position matching paradigms which combine passive proprioceptive stimuli with the visual space, and in which neither memory (e.g., as in remembered matching) nor planning and efference copy mechanisms (e.g., as in active matching) can play a role. There have been very few studies on gauge position matching, showing a slight tendency toward larger overestimation of larger angles (Gandevia et al., 2006; Smith et al., 2009), which is in line with the trend in the present study. However, they presented only a very limited number of different angles and these trends were not further quantified and discussed in the those papers. This could also be an indication for a more fundamental effect in the proprioceptive system in contrast to a setup-specific visual parallax error. As in the present experiment movements of different extents are presented, there exists the “constant duration vs. velocity” trade-off: either the duration or the velocity have to vary and add further confounds.

In the proposed assessment we opted for controlling the duration confound by choosing a constant movement duration of 1 s and varying the velocity, as perception of movement velocity—and thus kinaesthesia—is also a part of proprioception, whereas discrimination of time intervals between movement onset and end is not strictly related to proprioception. As a consequence, the present assessment paradigm is not a pure joint position sense test but also includes kinaesthesia. In our experiment the bell-shaped velocity profile of the passive minimum jerk movement was adapted in order to attain different movement extents. Thus, larger movements were performed with higher peak velocities (ranging from 18.8°/s for 10° to 56.2°/s for 30°). Whereas two active matching studies (Marteniuk et al., 1972; Goble and Brown, 2009) suggest that velocity has little influence on the accuracy of movement, respectively position, another study using a passive matching paradigm (Bevan et al., 1994) showed that increasing velocities result in larger constant errors resulting from an overestimation of position or movement. Furthermore, it was shown that the difference threshold of velocity perception, as well as variable errors in position increase with higher movement velocities (Bevan et al., 1994; Kerr and Worringham, 2002). Again, this demonstrates that such effects are very paradigm-dependent, thus worse estimates of larger movements in the present study could lead to an overestimation of position.

The complete understanding of the presented effect is beyond the scope of this paper and further experiments to elucidate the origin of over- and underestimation effects depending on the presented angle and velocity are recommended. They could include a larger range of angles and different sets of velocities or time intervals, as well as different initial positions.

### 4.3. Proprioceptive learning, duration and sex effects

The analysis of the proprioceptive outcome measures over the six measurements did not show systematic changes. Thus, the proprioceptive outcome measures appear to be robust against familiarity with the assessment and there was no substantial effect of proprioceptive learning. Furthermore, while variable, the time span between the two sessions had no effect on proprioceptive outcome measures. This supports the assumption that the proprioceptive wrist function of healthy young subjects remains stable over such periods of time and is not subject to systematic change. The duration of the assessments decreased from the first to the last measurement by up to 30%, suggesting an increasing level of familiarity with the task. Around 50% of the assessment time is required for the movements and new trial initiation and the remaining 50% fall upon the response time. Compared to the manual WPST which took approximately 5 minutes (Carey et al., 1996), our robotic implementation may have been able to reduce the assessment time because of two factors: fast and controlled generation of position stimuli, and feedback through a touchscreen. Instead of moving and aligning a physical protractor, it was sufficient to point on the touchscreen and the needle display would immediately jump to the provided position.

As in most studies investigating the effect of sex on proprioceptive matching errors in distal and proximal joints of the upper limb using different matching paradigms (Djupsjobacka and Domkin, 2005; Wycherley et al., 2005; Goble et al., 2006; Schmidt et al., 2013), no differences in proprioception between male and female subjects could be detected in this study. Yet, despite many studies showing concurrent results regarding the effect of sex, most studies (except the one by Schmidt et al., 2013 investigating passive elbow matching to a target position in 87 subjects) used rather small sample sizes, questioning the validity of these results. In contrast, a study on active contralateral matching involving elbow and shoulder with 209 subjects revealed an effect of sex on the absolute error (Herter et al., 2014). Thus, more large-scale studies with different matching approaches may be required to obtain conclusive results, and to investigate if the influence of sex on matching performance depends on the type of matching paradigm used.

### 4.4. Advantages and limitations of the automated gauge position matching paradigm

The gauge position matching paradigm presents a number of advantages over other matching paradigms. As the stimulus, i.e., presented angular joint position, is still present during the judging process, no memory is required as opposed to ipsilateral and contralateral remembered matching. It has been suggested that position sense asymmetries in contralateral remembered and concurrent matching tasks may arise from different limb/hemisphere-specific perception gains, i.e., a difference in the relationship between the passive displacement and the perception of the displacement (Adamo and Martin, 2009). Thus, in these paradigms the directional errors of the outcome may be biased by the non-tested limb/hemisphere. In the present gauge position matching experiment the task requires pointing to the perceived wrist orientation in a two-dimensional space and comparing the perceived orientation to the visual position of the gauge, rather than trying to match the proprioceptive information obtained from the tested limb and the non-tested limb, as in contralateral remembered and concurrent matching. Therefore, the proprioceptive information obtained from the respective movements leading to the end positions of the two limbs differ substantially and may not be used in direct comparison to perform the matching task. Furthermore, as the pointing limb and the gauge are not covered from the subject's sight while indicating and confirming the perceived position, visual feedback allows to reduce the confounding effect of proprioception of the non-tested limb, and to compensate for possible biases due to asymmetric proprioceptive gains and space representations, or interhemispheric transfer.

A further confound which can be avoided by using the gauge position matching paradigm is dependence on motor function potentially affecting movement replication and efference copies. This is especially important if the assessment is meant to be used in a clinical setting with patients which may suffer from motor deficits, as it is for example the case for 80% of stroke patients (Rathore et al., 2002). There are other completely passive paradigms such as the ipsilateral remembered matching implementations, where both the reference and the matching stimulus are presented passively and the subject is asked to provide a “stop” command when the positions match (Gay et al., 2010), or a recently proposed “ipsilateral concurrent matching” concept (Ingemanson et al., 2015) where the moment of overlap of two fingers doing passive crisscross movements has to be detected. However, both paradigms rely on reaction time. It can be argued that reaction delays could be compensated by movement prediction, as long as this prediction capability is not affected by a neurological injury or disease. The alignment of joint axis with the gauge and the viewpoint of the subject, which can lead to parallax errors in case of misalignment, presents the major challenge in the gauge position matching task. In the study evaluating the WPST in healthy subjects and stroke patients, adequacy of visual acuity and visuospatial skills was ascertained with a pretest wrist position, yet a well aligned head position was not enforced and thus parallax not controlled for (Carey et al., 1996). Since the gauge position matching paradigm combines proprioceptive space with visual space, modality-dependent space representations could play an important role, as demonstrated by the existence of a bias toward overshooting proprioceptive targets and undershooting visual targets suggesting a stretched proprioceptive space and contracted visual space (Adamovich et al., 1998; Goble and Brown, 2008; Goble et al., 2010). The underlying neurophysiological basis and whether this also affects the gauge position matching paradigm, however, remain yet unclear.

When comparing single-joint vs. whole-limb assessments, one could argue that assessments of whole-limb position sense (e.g., Dukelow et al., 2010) might better represent proprioceptive ability in real-world activities, such as reaching for an object. Furthermore, if adequate tools are used, whole-limb assessments can be useful to investigate differences in endpoint and joint errors (Herter et al., 2014), which could help understanding the processing of proprioceptive information from different muscles and joints. However, this requires complex and expensive setups in contrast to simpler devices requiring only one degree-of-freedom for single-joint assessments, which may be easier to introduce into a clinical setting (Hillier et al., 2015). Moreover, since there is some evidence for high agreement of somatosensory deficits in neighboring body parts after stroke (Connell et al., 2008), it may be redundant to assess multiple joints.

The presented robotic implementation of the WPST presents advantages over the manual application beyond better stimulus control and automation, such as accurate and precise sensing of position as well as automated calculation of different outcome measures and statistics. As the robotic device can render a wide range of environments (ranging from stiff position control to high transparency for free movements), it can also be used for all sorts of ipsilateral concurrent and remembered passive, active or gauge position matching tasks. Embedded high-resolutions torque and position sensors would allow to track active movements and interaction with the device over time to extract quantitative features. Furthermore, threshold tracking experiments using methods automatically adapting over a wide range of stimuli—such as in Rinderknecht et al. (2014), where a similar device was used for the metacarpophalangeal joint—can be implemented for more efficient and sensitive assessments. Thus, proprioceptive and motor function at the level of the wrist could be assessed in a more comparable way avoiding additional confounds due to different apparatuses.

### 4.5. Conclusion

In conclusion, the proposed robotic assessment provides reliable information, beyond absolute errors, on proprioceptive function of the wrist. This study showed that a sensitive tool providing continuous measurement outcomes could not only be used for basic research on proprioception, but shows also great potential for its application in clinical settings due to its rapid administration and simplicity. This would enable the clinicians to assess patients along the process of recovery and detect even minute changes for optimizing the individual rehabilitation process leading to clinically significant improvements. Furthermore, this paper highlights the importance of reporting (i) a comprehensive set of outcome measures—i.e., the different types of matching errors—, and (ii) reliability, its confidence interval and the methods used to compute them, to enable comparison of results across studies, whose outcomes vary significantly across publications.

## Author contributions

MR, WP, OL, and RG designed the study, MR and WP developed the methodology, MR performed the analysis, interpreted the results, and drafted the manuscript. MR, WP, OL, and RG revised the manuscript and approved the final version.

## Funding

This research was supported by the ETH Zurich Foundation in collaboration with Hocoma AG, and by the Janggen-Pöhn Foundation.

### Conflict of interest statement

The authors declare that the research was conducted in the absence of any commercial or financial relationships that could be construed as a potential conflict of interest.
